# The assessment and characterization of the built‐in internal photometer of primary diagnostic monitors

**DOI:** 10.1002/acm2.12047

**Published:** 2017-02-02

**Authors:** Andres E. Ruuge, Usman A. Mahmood, Yusuf E. Erdi

**Affiliations:** ^1^ Department of Medical Physics Memorial Sloan‐Kettering Cancer Center New York NY 10065 USA

**Keywords:** grayscale display function, liquid crystal displays, luminance, primary diagnostic monitor, quality assurance

## Abstract

The purpose of this work was to perform the initial evaluation of primary diagnostic monitor (PDM) characteristics following the implementation of New York City quality assurance (NYC QA) regulations on January 1, 2016, and compare the results of the QA measurements performed by an external photometer and the PDM manufacturer's built‐in photometer. TG‐18 and Society of Motion Picture and Television Engineers test patterns were used to evaluate monitor performance. Overall, 79 PDMs were included in the analysis. The verification of grayscale standard display function (GSDF) calibration, using a built‐in photometer, showed that only 2 out of 79 PDMs failed calibration. However, the same measurements performed by the external luminance meter showed that 15 out of 79 monitors had failed GSDF calibration. Measurements of the PDMs maximum luminance (L_max_), using an external photometer showed that 10 out of 53 PDMs calibrated for L_max_ = 400 cd/m^2^ and 17 out of 26 PDMs calibrated for L_max_ = 500 cd/m^2^ do not meet the manufacturer's recommended 10% tolerance limit for the target L_max_ calibration. Two PDMs did not pass the L_max_ ≥ 350 cd/m^2^ NYC QA regulations with L_max_ = 331 cd/m^2^ and L_max_ = 340 cd/m^2^. All tested PDMs exceeded the minimum luminance ratio (LR) of 250:1 as required by NYC QA regulations. Measurements taken of L_max_ and LR performed by a built‐in photometer showed that none of the PDMs had failed the NYC QA regulations. All PDMs passed the luminance uniformity test with a maximum nonuniformity of 17% (according to NYC regulations it must be less than 30%). The luminance uniformity test could only be performed using an external photometer. The evaluation of 79 PDMs of various ages and models demonstrated up to 18% disagreement between luminance measurements performed by the manufacturer's built‐in photometer when compared with those performed by an externally calibrated luminance meter. These disagreements were larger for older PDMs.

## 1 Introduction

The conformance of Primary Diagnostic Monitors (PDMs) used to make a final interpretation from images generated by radiological devices to the Digital Imaging and Communication in Medicine Grayscale Standard Display Function is increasingly required by several state and city regulators. For example, effective from 1 January 2016, all radiological equipment registrants in New York City (NYC) are required to extend their Quality Assurance (QA) program to their PDMs. This work describes our experience in implementing the PDM performance and conformance with the NYC QA program^1^ and characterization of the luminance measurement performance of the internal, built‐in photometer of Barco monitors against an externally calibrated luminance meter.

## 2 Methods

During the on‐site annual testing of PDMs, several luminance characteristics were evaluated, including GSDF conformance, maximum luminance (L_max_), luminance ratio (LR), and luminance uniformity.

Measurements were performed on 79 PDMs: color display system models MDCC‐6130 (41), MDCC‐6230 (9), MDCC‐6330 (1) and Grayscale display system models MDCG 3120‐CB (2), MDCG‐10130 (2), MFGD 3420 (6) and MFGD 5421 (18) (Barco; Duluth, Georgia) as part of annual QA testing following the NYC regulations.^1^ Monitors were located at various hospital sites, radiology physicians’ offices, and radiology reading rooms. All measured PDMs were liquid crystal displays equipped with the manufacturer's built‐in photometers (Barco, I‐Guard) and connected to Barco MediCal QAWeb service for manual and automatic quality control measurements. PDM combinations included 1, 2, and 4 monitors, depending on the location. TG‐18 and Society of Motion Picture and Television Engineers test patterns were used to evaluate monitor performance. An externally calibrated photometer (RaySafe Solo Light; Billdal, Sweden) was used to measure the luminance values.

### 2.A Measurement and verification of Grayscale standard display function

For each PDM, the luminance response is expressed as the change in luminance between gray levels (dL/L) for the just noticeable difference plotted against the digital driving level (DDL).^2^, ^3^ To validate the results of GSDF calibration that was performed using the built‐in photometer, TG18LN1‐18 luminance patterns were measured with an external photometer. All measurements were performed under normal reading conditions. NYC QA guidelines^1^ and AAPM Task Group 18^4^ require 10% tolerance for GSDF calibration.

### 2.B Maximum luminance and luminance ratio

The perceived contrast characteristics of an image depend on the ratio of maximum luminance L_max_ (all white) to minimum luminance L_min_ (all black).^3^ Luminance values were measured with an external photometer using TG18‐LN01 and TG18‐LN18 test patterns. Following NYC regulations^1^ and the ACR technical standard,^5^ the maximum luminance L_max_ of nonmammography PDMs should be at least 350 cd/m^2^. The LR was calculated using the following equation:

(1) $$\text{LR}=L_{\max}/L_{\min}$$

where LR is the luminance ratio, L_max_ is the maximum luminance, and L_min_ is the minimum luminance.

### 2.C Evaluation of luminance uniformity

Using an external photometer, luminance values were measured at five locations on the PDM: the center and at each quadrant using the TG18‐UNL80 test pattern. The luminance uniformity (LU) of a display was calculated using the following equation:

(2) $$\text{LU}=\left(L_{2}-L_{1}\right)/\left(L_{2}+L_{1}\right)\times 200$$

where L_2_ is the maximum and L_1_ is the minimum measured luminance of the quadrants and the center.

Because the built‐in photometer can measure luminance only at a fixed location (the bottom right corner for PDM models MDCG 3120‐CB, MFGD 3420, and MFGD 5421 and the top middle portion of the screen for PDM models MDCC‐6130, MDCC‐6230, MDCC‐6330, and MDCG‐10130), a LU test is performed only with an external photometer.

### 2.D Ambient light measurements

A RaySafe Solo Light photometer used in the study was equipped with the clip‐on cover that eliminates the effect of ambient light on luminance measurements. All measurements of the luminance test patterns are performed with direct contact of the photometer with the measured PDM. The ambient luminance values were measured as a reflected ambient light from the screen of a turned off monitor (one foot away from the center of the monitor). These measurements were used to adjust the monitor GSDF calibration using the formula:

(3) $$L_{\mathrm{adjusted}}=L_{\mathrm{meas}}+L_{\mathrm{amb}}$$

where L_meas_ is a measured luminance and L_amb_ is a measured ambient luminance.

In addition to luminance measurements, ambient light was measured to confirm that the monitors did not receive outside light flow and they provide reading conditions mentioned in the ACR manual.^5^

Illuminance was measured using the same RaySafe light photometer with the illuminance sensor positioned in the center of the display facing outward while the PDM was turned off. Illuminance values are used to estimate normal reading conditions (20‐40 Lux).

## 3 Results

### 3.A Grayscale standard display function

**Figure 1:**
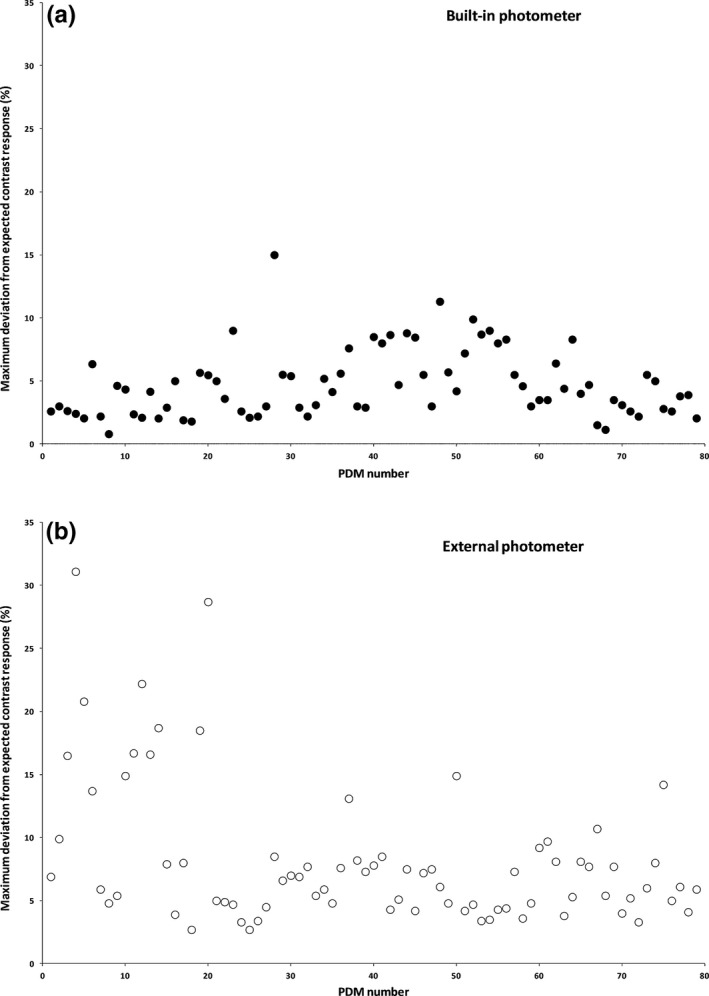
The GSDF conformance tests performed by the built‐in Barco luminance meter (a) and by an external RaySafe photometer (b). For the built‐in luminance meter, the measured luminance values for each digital driving level were within the 10% tolerance limit for 77 monitors and failed for two monitors (15% and 11%). Measurements performed by the external luminance meter showed that 15 out of 79 monitors had failed the 10% tolerance limit within the range of 10.7% and 31.1%. The average deviation of contrast response for all 79 monitors 8.2% ± 5.6%.

Figure 1 represents the results of GSDF conformance tests performed automatically by the built‐in luminance meter (a) and by an external photometer (b). For the built‐in luminance meter, the measured luminance values for each DDL were within the 10% tolerance limit for 77 monitors and failed for two monitors (15% and 11.3%). Measurements performed by the external luminance meter showed that 15 out of 79 monitors had failed the 10% tolerance limit within the range of 10.7% and 31.1%. The average deviation of contrast response for all 79 monitors is 8.2% ± 5.6%.

### 3.B Maximum luminance and luminance ratio

**Figure 2:**
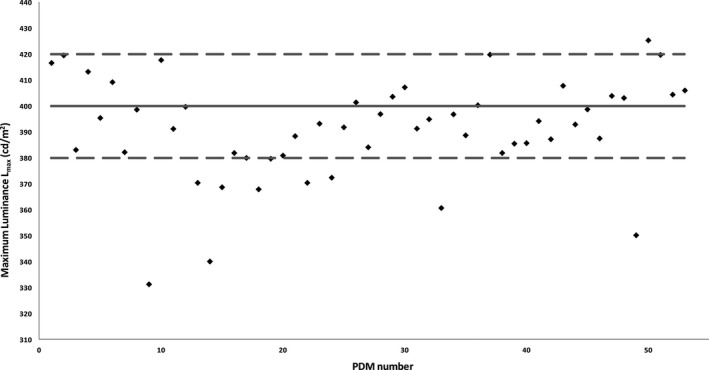
L_max_ values measured with an external photometer for the PDMs with target calibration for L_max_ = 400 cd/m^2^. Measurement results show that 10 out of 53 PDMs calibrated do not meet the manufacturer's recommended 10% tolerance limit for the target L_max_ calibration. Two out of 79 monitors did not pass the L_max_ ≥ 350 cd/m^2^ NYC QA regulations.

**Figure 3:**
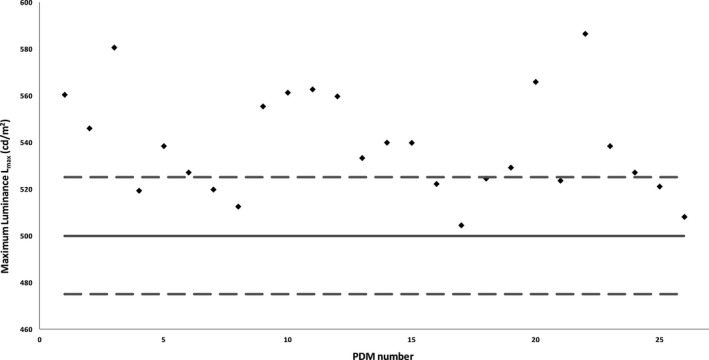
L_max_ values measured with an external photometer for the PDMs with target calibration for L_max_ = 500 cd/m^2^. Results show that 17 out of 26 PDMs do not meet the manufacturer's recommended 10% tolerance limit for the target L_max_ calibration.

Figures 2 and 3 represent the L_max_ values measured with an external photometer for PDMs with a target calibration of L_max_ = 400 cd/m^2^ (Fig. 2) and L_max_ = 500 cd/m^2^ (Fig. 3). Measurement results showed that 10 out of 53 PDMs with a target calibration of L_max_ = 400 cd/m^2^ and 17 out of 26 PDMs with a target calibration of L_max_ = 500 cd/m^2^ did not meet the manufacturer's recommended 10% tolerance limit for the target L_max_ calibration. Two out of 79 monitors did not pass the L_max_ ≥ 350 cd/m^2^ NYC QA regulations with values of L_max_ = 331 cd/m^2^ and L_max_ = 340 cd/m^2^. The difference between built‐in and external photometer luminance measurements for L_max_ was 5.1% ± 4.2% for all tested PDMs.

**Figure 4:**
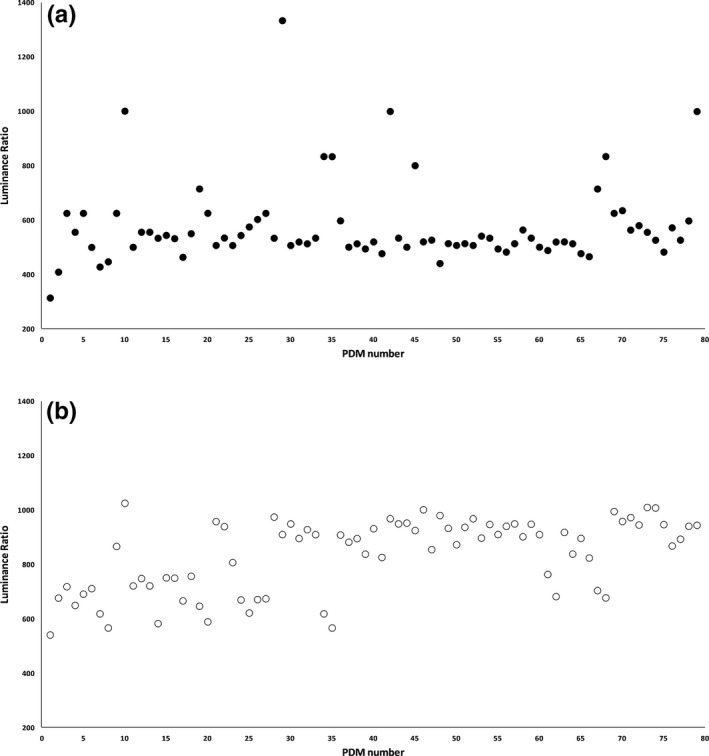
Results of luminance ratio measurements performed by a built‐in photometer (a) an external luminance meter (b). All tested PDMs exceeded the luminance ratio of 250:1 as required by NYC PDM guidelines.

Figure 4 shows the results of LR measurements performed by a built‐in photometer (a) and an external luminance meter (b). All tested PDMs exceeded the luminance ratio of 250:1 as required by NYC PDM guidelines.

### 3.C Evaluation of luminance uniformity

**Figure 5:**
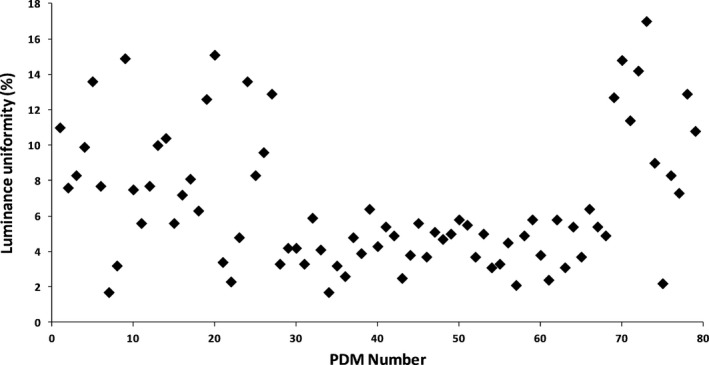
Luminance uniformity measurements performed by an external RaySafe photometer. All PDMs passed the luminance uniformity test, which is 30% according to NYC regulations. Barco PDM model MDCC‐6130 had luminance nonuniformity less than 6%. PDMs manufactured between 2009 and 2012, and after 2013, showed luminance nonuniformity up to 17%.

Figure 5 represents the results of luminance uniformity measurements performed by an external photometer. All PDMs passed the luminance uniformity test, which was 30% according to NYC regulations. Luminance nonuniformity up to 17% was observed in PDMs connected in 2009 through 2016. The Barco MDCC‐6130 model connected in 2012 and 2013 had luminance nonuniformity values less than 6%.

### 3.D Ambient light and illuminance measurements

The mean value of illuminance measured with an external meter was 6.2 lux ± 3.6 lux. The mean ambient luminance was 0.19 cd/m^2^ ± 0.11 cd/m^2^. These levels are well below the ACR recommended 20 lux for a nonmammography diagnostic monitor radiology reading room.^5^

## 4 Discussion

The evaluation of Barco primary diagnostic monitors of various ages and models demonstrated a discrepancy between luminance measurements performed by the manufacturer's built‐in photometer when compared with those performed by an externally calibrated luminance meter. This discrepancy was larger for older PDMs, confirming the findings of Silovsky and Marsh.^6^ However, the number of PDMs manufactured after 2014 also showed a disagreement in luminance values measured by external and built‐in photometers. All tested PDM models had a built‐in photometer sensor positioned either at the bottom right corner (models MDCG 3120‐CB, MFGD 3420, and MFGD 5421) or at the top middle part of the screen (MDCC‐6130, MDCC‐6230, MDCC‐6330, and MDCG‐10130). Figure 5 represents the luminance uniformity for all tested PDM models, sorted by the age of the monitor, where the first monitor is Barco MFGD 5421 (September 2009) and the 79th monitor is Barco MDCC 6330 (January 2016) (See Table 1). Barco MDCC‐6130 monitors (21–23, 28–33, 36–66, and 75) showed the lowest nonuniformity values, between 2.1% and 6.4% with a mean value of 4.26% and standard deviation of 1.21%. Table 2 summarizes the luminance uniformity and the difference in the maximum luminance measurements between the built‐in and external photometers for the Barco PDM model MDCC‐6130. There is no direct dependence between PDM uniformity and the difference in luminance measurements for the external and built‐in photometers as can be observed.

**Table 1:** List of all measured Barco PDM models included in the study and sorted by the year of first use

#	Model	In use since	#	Model	In use since	#	Model	In use since
1	MFGD 5421	09/2009	28	MDCC‐6130	03/2012	55	MDCC‐6130	01/2014
2	MFGD 5421	09/2009	29	MDCC‐6130	05/2012	56	MDCC‐6130	01/2014
3	MFGD 5421	10/2009	30	MDCC‐6130	05/2012	57	MDCC‐6130	01/2014
4	MFGD 5421	10/2009	31	MDCC‐6130	05/2012	58	MDCC‐6130	02/2014
5	MFGD 5421	10/2009	32	MDCC‐6130	05/2012	59	MDCC‐6130	03/2014
6	MFGD 5421	10/2009	33	MDCC‐6130	05/2012	60	MDCC‐6130	03/2014
7	MDCG 3120‐CB	11/2009	34	MFGD 3420	06/2012	61	MDCC‐6130	03/2014
8	MDCG 3120‐CB	11/2009	35	MFGD 3420	06/2012	62	MDCC‐6130	03/2014
9	MFGD 3420	11/2009	36	MDCC‐6130	09/2012	63	MDCC‐6130	03/2014
10	MFGD 3420	11/2009	37	MDCC‐6130	10/2012	64	MDCC‐6130	03/2014
11	MFGD 5421	11/2009	38	MDCC‐6130	11/2012	65	MDCC‐6130	03/2014
12	MFGD 5421	11/2009	39	MDCC‐6130	11/2012	66	MDCC‐6130	03/2014
13	MFGD 5421	11/2009	40	MDCC‐6130	08/2013	67	MDCG‐10130	05/2014
14	MFGD 5421	11/2009	41	MDCC‐6130	08/2013	68	MDCG‐10130	05/2014
15	MFGD 5421	11/2009	42	MDCC‐6130	08/2013	69	MDCC‐6230	06/2014
16	MFGD 5421	11/2009	43	MDCC‐6130	08/2013	70	MDCC‐6230	09/2014
17	MFGD 5421	11/2009	44	MDCC‐6130	08/2013	71	MDCC‐6230	09/2014
18	MFGD 5421	11/2009	45	MDCC‐6130	08/2013	72	MDCC‐6230	11/2014
19	MFGD 3420	06/2011	46	MDCC‐6130	08/2013	73	MDCC‐6230	11/2014
20	MFGD 3420	06/2011	47	MDCC‐6130	08/2013	74	MDCC‐6230	11/2014
21	MDCC‐6130	03/2012	48	MDCC‐6130	08/2013	75	MDCC‐6130	12/2014
22	MDCC‐6130	03/2012	49	MDCC‐6130	12/2013	76	MDCC‐6230	06/2015
23	MDCC‐6130	03/2012	50	MDCC‐6130	12/2013	77	MDCC‐6230	06/2015
24	MFGD 5421	03/2012	51	MDCC‐6130	01/2014	78	MDCC‐6230	06/2015
25	MFGD 5421	03/2012	52	MDCC‐6130	01/2014	79	MDCC‐6330	01/2016
26	MFGD 5421	03/2012	53	MDCC‐6130	01/2014			
27	MFGD 5421	03/2012	54	MDCC‐6130	01/2014			

**Table 2:** Luminance uniformity and maximum luminance values for the PDM Barco MDCC 6130 measured by external and built‐in photometers

#	L_max_ external meter	L_max_ built‐in meter	Nonuniformity	Difference for L_max_	#	L_max_ external meter	L_max_ built‐in meter	Nonuniformity	Difference for L_max_
	(cd/m^2^)	(cd/m^2^)	(%)	(%)		(cd/m^2^)	(cd/m^2^)	(%)	(%)
1	383.2	400.6	3.4	4.3	22	372.5	401.0	4.7	7.1
2	413.3	400.9	2.3	3.1	23	391.9	400.9	5.0	2.2
3	395.5	400.4	4.8	1.2	24	401.5	400.7	5.8	0.2
4	409.3	400.3	3.3	2.2	25	384.2	400.7	5.5	4.1
5	382.3	400.0	4.2	4.4	26	397.0	400.6	3.7	0.9
6	398.7	400.5	4.2	0.5	27	403.7	400.7	5.0	0.8
7	331.4	400.3	3.3	17.2	28	407.3	400.4	3.1	1.7
8	417.8	400.4	5.9	4.4	29	391.4	400.4	3.3	2.2
9	391.3	400.6	4.1	2.3	30	395.0	400.6	4.5	1.4
10	399.8	400.5	2.6	0.2	31	360.8	400.6	2.1	9.9
11	370.5	400.8	4.8	7.6	32	396.9	400.5	4.9	0.9
12	340.2	400.3	3.9	15.0	33	388.8	400.7	5.8	3.0
13	368.8	400.5	6.4	7.9	34	400.4	400.7	3.8	0.1
14	382.0	400.6	4.3	4.6	35	419.9	400.7	2.4	4.8
15	380.1	400.7	5.4	5.1	36	382.0	400.3	5.8	4.6
16	368.0	399.8	4.9	8.0	37	385.6	400.6	3.1	3.8
17	379.8	400.6	2.5	5.2	38	385.8	400.4	5.4	3.7
18	381.0	400.6	3.8	4.9	39	394.3	400.8	3.7	1.6
19	388.5	400.2	5.6	2.9	40	387.3	400.7	6.4	3.3
20	370.5	400.7	3.7	7.5	41	350.3	400.6	2.2	12.6
21	393.3	400.3	5.1	1.8					

However, as indicated in Table 2, built‐in photometer measurements appear to be remarkably consistent across a large number of monitors even though the external photometer measurements vary significantly. Because PDMs are automatically calibrated by the system software, this software uses an internal photometer calibration value of 400 cd/m^2^. Therefore, in the event that the luminance value of a PDM fluctuates from the calibration value, the internal photometer will always read luminance values close to 400 cd/m^2^ because that is the calibration point. To test this, additional tests were run where the luminance value of the same monitor using four different external photometers (RaySafe Solo Light, RaySafe Xi, Konica–Minolta CA‐210, and Konica‐Minolta CS‐100A) was measured. The results showed that luminance values varied 0.8% between these photometers (the results are not presented here). In light of this test, we speculate that external measurements are closest to an actual luminance value, whereas internal measurement is biased to the PDM calibration level.

One area of future work is to examine the effect of monitor warm‐up time on the maximum luminance measurement by external and built‐in photometers. As a small pilot study, 5 Barco MDCC‐6130 PDMs located in the same radiology reading room were measured under the same conditions. The mean change in maximum luminance measurements during the first 30 min after the monitor was turned on (following a 12‐hour turned off period), was 12.7 cd/m^2^ ± 7.6 cd/m^2^ for the measurements performed by an external photometer and 0.9 cd/m^2^ ± 0.5 cd/m^2^ for those performed by the built‐in photometer. This shows the sensitivity of an external photometer to PDM warm‐up period and confirms the importance of performing the PDM QA measurements after at least a 30‐minute warm‐up time for the monitor. We plan to examine the possible effect of the PDM's temperature during GSDF calibration on its performance and luminance characteristics.

## 5 Conclusion

We have investigated internal and external photometer performance of PDMs and have found that there are many more monitors that fail external photometer luminance measurements compared with those measured with an internal photometer when tested according to NYC PDM guidelines.

The discrepancy between the luminance measurement by external and built‐in photometers should be followed up with the hospital IT department and manufacturer shortly after PDM testing.

## Conflict of interest

None.
